# Early Steps in Automated Behavior Mapping via Indoor Sensors

**DOI:** 10.3390/s17122925

**Published:** 2017-12-16

**Authors:** Taner Arsan, Orcun Kepez

**Affiliations:** 1 Department of Computer Engineering, Kadir Has University, 34083 Istanbul, Turkey; 2 Department of Interior Architecture and Environmental Design, Kadir Has University, 34083 Istanbul, Turkey; orcun.kepez@khas.edu.tr

**Keywords:** sensors and technologies for indoor localization systems, positioning strategies and algorithms, behavior mapping, activity monitoring, ultra-wide band sensors

## Abstract

Behavior mapping (BM) is a spatial data collection technique in which the locational and behavioral information of a user is noted on a plan layout of the studied environment. Among many indoor positioning technologies, we chose Wi-Fi, BLE beacon and ultra-wide band (UWB) sensor technologies for their popularity and investigated their applicability in BM. We tested three technologies for error ranges and found an average error of 1.39 m for Wi-Fi in a 36 m^2^ test area (6 m × 6 m), 0.86 m for the BLE beacon in a 37.44 m^2^ test area (9.6 m × 3.9 m) and 0.24 m for ultra-wide band sensors in a 36 m^2^ test area (6 m × 6 m). We simulated the applicability of these error ranges for real-time locations by using a behavioral dataset collected from an active learning classroom. We used two UWB tags simultaneously by incorporating a custom-designed ceiling system in a new 39.76 m^2^ test area (7.35 m × 5.41 m). We considered 26 observation points and collected data for 180 s for each point (total 4680) with an average error of 0.2072 m for 23 points inside the test area. Finally, we demonstrated the use of ultra-wide band sensor technology for BM.

## 1. Introduction

Behavior mapping is an outcome of systematic observation and provides significant design information through linking spatial attributes to the behaviors of the people observed [1,2]. The observation of human behavior requires an interface to represent the use of different settings and objects through their location and proximity. Behavior maps provide the locations of people (and other observed features) in which one can assess the occupancy of the space, the use of features within that space and social interaction based on the proximity between, and the orientation of, people in a group. Behavior mapping serves as an innovative way of understanding the relationship between the environment and human behavior.

A single behavior map ideally consists of a scaled plan layout of the studied area on which the locations of people and their behaviors being measured by the conducted study are marked [3]. A systematic observation is comprised of observation cycles in which the time interval between each cycle, which is based on the behaviors studied, is equal. The researcher produces a behavior map for each observation cycle. After several behavior maps are collected on a plan layout, they are overlapped to make an overall map. This overall map is an important source for understanding behavioral patterns in a given space, since it provides visual data about the observed activities [4]. By consulting these overall maps, design researchers and practitioners can start to question why some settings are used more often than others, how the environment being studied works for different user groups throughout the day and the relationship between design features and the behaviors they support. These questions are often connected to theories used for understanding the environment and behavior [1,5].

Behavior mapping as a method has been used in studies of both indoor and outdoor environments. One of the earliest studies conducted on indoor spaces utilized behavior mapping to show differences in social interaction between patients in single-, double- and multiple-occupancy rooms [6]. Moore used behavior mapping to study the differences in the use of the outdoors, with emphasis on differences in the outdoor behavior of girls and boys after the reconstruction of a playground [7]. The method was widely used in studying outdoor settings such as outdoor children’s museums and neighborhood parks due to its unobtrusive applicability in public spaces [4]. Indoor applications of this method in design research, on the other hand, have been fairly limited, with those having been carried out focusing largely on seniors housing [8,9,10].

The term behavior mapping is sometimes used in reference to pre-designed tables, which are prepared for systematic observation and which include a list of space names and behavior codes to be observed [11]. Often referred to as a behavioral table or a behavior mapping matrix, this data collection method does not include a plan layout for recording people’s locations. A literature review carried out in the mid-1980s indicated that the use of behavioral tables for data collection with regard to indoor settings was a popular method at that period [11]. In work on hospitals, behavioral tables were adopted instead of a plan layout [7]. Similarly, in more recent research on hospitals, behavioral tables have been preferred for recording data during observation cycles [12,13]. The main reason for the use of tables rather than a plan layout for data collection in these studies can be attributed to researchers’ own preferences. As non-designers, it is possible that they were more familiar with collecting data using tables instead of plan layouts. Moreover, some of the most popular research software for behavior mapping, mostly used by researchers in other disciplines, codes the spatial location superficially; in other words, when this software is used, the “location” is reduced to merely the name tag of the observed area.

Cosco et al. proposed using the behavior mapping technique along with other established instruments for a similar purpose for physical activity research in outdoor children’s playgrounds [14]. Unlike behavior mapping, the aforementioned, established observation instruments require training for observers and include certain measures intended to maintain inter-rater reliability and validity [15,16]. The power of behavior mapping lies in its ability to reflect the connections between elaborative features of the environment and observed behavior. However, issues with establishing the instrument’s validity may hinder its adoption by disciplines other than design.

Traditionally, the behavior mapping technique used hand-written records known as the paper-pencil method. With Van Andel’s research [17], in which behaviors and attributes of the built environment in outdoor settings were recorded digitally, we see the initial efforts to introduce the use of digital means to this technique. Early on, time-lapse photography and video technology were also used while documenting observed behaviors [3]. More recently, digital devices, such as personal digital assistants (PDA) and tablets, have made it possible to record more variables in a shorter amount of time [4]. Studies at the time, which were mostly conducted outdoors, began to incorporate geographical information systems (GIS) for behavior mapping. Although the inclusion of GIS technologies followed a procedure similar to the way researchers manually collected data with a paper and pencil, it also offered the possibility of using powerful data analysis tools [4,18]. Thus, despite utilizing digital technologies during analysis, the data collection procedure still has room for manual methods for data collection through the use of the traditional “pencil on paper” method [8,10,19]. Due to methodological issues and the researcher bias mentioned above, Sommer and Sommer stated that behavior mapping can be time and resource consuming [20]. Although there are new technologies used for behavior mapping, they do not really offer any innovation in data collection: instead, they provide tools for data management and visualization.

Some other disciplines that use observation in the field to record behaviors have adopted technological innovations rapidly to cope with problems associated with the technique. In his review of the uses of field-based observation instruments to measure physical activity, McKenzie concluded that “these techniques are underused” for various reasons [15]. In parallel to this argument, much valuable effort has been expended in tracking outdoors travel behavior automatically by using the Global Positioning System (GPS). Cycling and walking activities have been tracked successfully by the use of GPS technologies [21,22]. GPS has also been employed to study the role of the built environment in obesity [23]. The use of GPS along with accelerometers has provided invaluable information about features of the urban environment and physical activity, without the burden of exhaustive field research. Although GPS tracking is limited to the outdoors, the improvement it brings to field research by eliminating observation has also inspired researchers to look for similar alternatives that would work indoors. This study aims to test candidate technologies, which can be used to determine individuals’ locations with adequate accuracy to allow behavior mapping indoors. Earlier efforts in indoor positioning remain at the level of specific applications for a given problem rather than linking the indoor environment to behavior.

Our literature review has shown that the adoption of this tool goes well beyond the design research and has several applications that inform disciplines including medicine, nursing and exercise sciences. It can be stated that behavior mapping is a holistic and inclusive tool providing complex information that can be analyzed in accordance with different research problems. By employing behavior mapping, one can inquire into and discover patterns of behavior that may otherwise not be possible and develop research-based insights for innovation. Yet, due to its time-consuming protocol for the collection of data and its questionable reliability due to human error, behavior mapping does not receive the reputation it deserves as a field technique. On the other hand, indoor positioning technologies solve the problems associated with behavior mapping by removing the presence of an observer and providing reliable data. However, real-world applications of indoor location positioning are often built around well-defined engineering problems and overlook the complexity of the spatial information collected. Thus, they reduce people’s locations to the position of tags in interior space and do not study the complex patterns of people’s natural movement, the self-selection of spaces to occupy, the self-selection of the use of tagged objects, the formation of groups or social interaction based on proximity between people. The use of sensors for automated behavior mapping will fill an important gap, marrying the engineering fields that study indoor positioning and other disciplines that employ an understanding of human behavior for decision-making.

This study first reviews the literature on behavior mapping and then reviews the literature of indoor positioning systems with emphasis on technologies selected to be further studied in the scope of this paper. The contribution of the study is three-fold. Firstly, it broadens the understanding of indoor location positioning from the level of a well-defined engineering problem to that of a tool that serves as a visual database to enquire into a complex set of questions regarding the time spent in different locations, the use of objects and the social interaction between people based on proximity. Secondly, it employs a real behavioral dataset to demonstrate how these technologies would work for behavior mapping if selected. Thirdly, we produced a behavior map through employing UWB sensor technology. 

## 2. Testing Candidate Technologies for Behavior Mapping of the Indoors

“Real-time location system” (RTLS) is the most common name for the technologies used for detecting a current geographic location. An RTLS consists of wireless nodes that send and receive signals and process the signals received. Current RTLS are based on wireless technologies, such as infrared positioning, ultrasonic positioning systems, radio frequency identification (RFID), Wi-Fi, Bluetooth and ultra-wide band (UWB), and are based on the other technologies including, but not limited to, long-range capacity sensing [24], pressure on the floor or air, acoustic, infrared and light (camera and image processing) [25]. A comparison of RTLS and behavior mapping (BM) can be found in Table 1. Two extensive reviews conducted in the last decade have been pointing out challenges and opportunities of RTLS as summarized in Table 1 [25,26]. Our literature review also illuminated challenges and opportunities of behavior mapping through critical analysis.

### 2.1. Technologies for Use in Indoor Positioning-Related Work

The Global Positioning System (GPS) is a technology that is both suited to and efficient for outdoor spaces [27]. If the position detection is performed outdoors, it is carried out using a GPS signal provided by the satellites or an assisted-GPS signal provided by the Global System for Mobile Communications (GSM) operators. The average error is one to five meters. For outdoor locations, GPS is the most widely-used location and tracking technology in the world [27]. If indoor positioning is considered, then position detection can be performed via infrared, ultrasonic, cellular, RFID, Wi-Fi, Bluetooth or UWB sensors. In this case, depending on the technology used, the average error can be reduced from the meter level to the centimeter level. Before providing the details of the current study, a brief overview of existing indoor positioning systems is necessary.

Active badges were the first indoor positioning system developed by AT&T Cambridge [28]. In this system, each employee wears a small device that transmits a unique infrared. A central database collects the data from the infrared sensors and, thanks to the RF tags worn by each user, allows the location of all users to be determined. However, this technique can only be used for short-range communications. Moreover, the infrared technique requires a line of sight (LOS) between the transmitter and the receiver.

AT&T Cambridge then developed an ultrasonic tracking technology named Active Bats, which provided better accuracy than active badges. In this system, the user wears small badges that emit ultrasonic pulses for the transmitter [29]. The system uses a triangulation algorithm and measures the time-of-flight (ToF) of this pulse from the transmitter to a known point in the ceiling. With this measurement, the distance between bats to each receiver can be calculated since the speed of sound in air is known. The implementation of the active bats system is quite difficult due to the high number of transmitters that must be installed and the fine-tuning they require.

RFID technology stands out due to having a lower cost than ultrasonic positioning systems. It is an automatic identification technology that uses radio signals to track and to estimate the exact locations of people or “objects” like devices and vehicles. In this system, objects have RFID tags, which are identified using an RFID reader [30,31]. These tags have unique ID numbers for the purposes of identifying which reader is interrogating a tag. When these unique tag IDs are associated with the people who carry them, the location of individuals can be detected.

RFID has been a reliable technology for selective object identification. Recently, Bluetooth low energy (BLE) beacons and UWB sensor technologies have been used as an alternative option for tracking and indoor positioning [32]. Bluetooth networking communicates on a frequency of 2.45 gigahertz. BLE beacons have a reachability range of about 15 m, which is much wider than those of an RFID sensor. The use of received signal strength indications (RSSI) is recommended to help with positioning [33]. As the distance between the sender and receiver decreases, the RSSI value decreases, as well. Trilateration is a method of estimating the locations of points by distance measurements, using the geometry of a minimum of three transmitters [34]. The traveler’s position is determined as the point of intersection of three circles, each centered on one of the BLE beacons. Performance analysis shows accuracy that is within five meters. This is not sufficient for indoor positioning, but can be acceptable when providing directions and routing [34].

Wi-Fi (to IEEE 802.11 standards) is the most popular technology used for wireless communication today. Wi-Fi operates on different bands including 2.4 GHz and has a coverage range of 50 to 100 m [33]. Most electronic devices, including computers and mobile phones, have Wi-Fi adapters. Moreover, almost all public buildings (e.g., universities, hospitals, etc.) have wireless local area network (WLAN) infrastructures. Since this technology is already a part of our daily life, it can be used for estimating location in an indoor environment. Within the WLAN system, wireless access points (APs) act as the stations that transmit and receive data. Multiple APs can serve multiple users; when a user moves beyond the access point’s range, they are automatically handed over to the next one. RSSI is a value that indicates strength when the propagated radio wave is received. In order to determine the relationship between RSSI and distance, AP is kept at a fixed known point, and RSSI are collected while slowly moving away from the transmitter. Time-of-arrival (ToA) measures the round-trip time (RTT) between the signals of transmitter and receiver [35]. The RTT basically measures the total time of the signal traveled between the source and destination. The Euclidean distance between transmitter and receiver can be calculated by the multiplication of travel time by wave speed [36]. ToA requires clocks on the transmitter and receiver to be very accurate and synchronized since measurement accuracy is crucial when determining location accuracy.

Ultra-wide band (UWB) is a radio technology for short-range high-bandwidth communication. UWB can be ideal for indoor distance estimation, localization and tracking [37]. UWB has a very large bandwidth, greater than 500 MHz, and for this reason, signals often arrive at the receiver via multiple paths. However, its large bandwidth also allows for diverse frequencies to be used at different times, which can be used as a solution to multipath problems and interference [38]. UWB transmitters consumes a low amount of power in comparison with other positioning technologies, allowing better power efficiency and a longer battery life for UWB devices than other options. The power consumption of UWB transmitters that are worn by people is generally less than 1 mW, whereas the power consumptions of UWB receivers are around 400 mW [39]. The UWB frequency range for communication applications is 3.1 to 10.6 GHz in the Federal Communications Commission (FCC)-approved UWB band [40]. This eliminates the need for an additional 2.4-GHz radio link and operates only in the FCC-approved UWB band [41]. In the presence of other people and objects, the LOS path can be blocked, causing the initial direct path to be mapped with a delay and, thus, causing a bias. It is natural to expect non-line-of-sight-based errors, yet it is hard to conclude that the absorbent effects of the human body will increase these errors [42,43]. ToF and ToA measurements are used for precise indoor positioning with an average error of less than 0.30 m. Apart from a few industrial implementations, UWB has not entered the mass market because it requires a dedicated transmitter and receiver infrastructure [36]. The use of UWB sensors with the time difference of arrival (TDoA) has received particular attention in medical applications, with an accuracy of 3 to 5 mm [41,44,45]. The angle of arrival (AoA) technique compares an estimation of the angles from which signals are received from at least two sources with the amplitude of the signal. The location of the object can be found from the angle at which the signals intersect [46]. The AoA technique can be used together with other techniques as a hybrid method to increase its accuracy [47].

Zhang et al. used UWB sensors and accelerometers to understand the use of workstations in a non-territorial office space [48]. Yet, this study used predetermined zones to embed sensors to understand use. Wearable UWB sensors carried by employees provided dichotomous (zero and one) information whenever they were detected in the zone. An open office plan layout was divided into several zones to understand the use of each zone by different groups of employees. This is similar to a type of behavior mapping where use of a specific place is recorded on a tally sheet with a name tag. However, this study did not report accuracy as a concern, since researchers were interested in the headcount of the type of users in predetermined zones. Thus, this effort employing UWB technology to understand location in an indoor setting did not accurately determine the indoor location of an individual, but reported the use of a predetermined zone. This application is based on an older type of behavior mapping that took only the name of the used space into consideration and that cannot be regarded as predecessor of our study.

In order to carry out the automated behavior mapping, it is necessary to specify the indoor positioning technologies to be used. Thus, this research focused on the three particular technologies that have been selected as the most likely candidates for achieving the highest rate of accuracy with the least intervention to the indoor design. First Wi-Fi, then BLE beacon and, finally, ultra-wide band (UWB) sensor infrastructures were installed, and various tests were conducted. A comparison of the selected indoor positioning technologies is given in Table 2.

### 2.2. The Methodology and the Test Results

In our earlier pilot experiments, we had installed Wi-Fi, Bluetooth low energy beacons and the ultra-wide band sensor infrastructures in the test areas that we set up at the university campus. We conducted several accuracy measurement tests with each of these technologies, all of which used variations of RSS-based lateration algorithms. We implemented lateration with the least squares algorithm, as described in [49]. We assume that there are three access points AP_1_, AP_2_ and AP_3_. These access points are located at the ($x_{1},y_{1})$, ($x_{2},y_{2})$ and ($x_{3},y_{3})$ coordinates, respectively. The RSS measurements can be converted into distance values. Typically, the RSS values are measured in dBm and usually range between 0 dBm (excellent signal) and –100 dBm (poor signal). A decrease is observed in the RSS values as the distance between the transmitting and measuring devices increases. It is possible to show the relationship between the transmitted and received power with respect to the distance between the transmitting and receiving devices [33]. Therefore, after measuring the received power at the target location, the distance of a target from the transmitter (APs) can be calculated. As we mentioned in [49], we used the following equation to compute the RSS indication (RSSI) specified by [50]:

(1) $$RSSI=-\left(10\;n\;log_{10}d+A\right)$$

where *n* is signal propagation constant, *d* is the distance from the sender and *A* is the received signal strength at a distance of one meter. Then, the linear RSSI equation can be obtained as:

(2) $$n_{i}=-\left(\frac{RSSI_{i}-A}{10\;log_{10}d_{i}}\right)$$

The RSSI values are then converted to distances by considering the calibrated propagation constant. As noted earlier, the RSS measured values are affected by the position of the Wi-Fi antennas, the construction materials of the building and the walls. Thus, in order to overcome these handicaps, we utilized data fitting by least squares in order to increase the accuracy of the algorithm.

Here, we first present the Wi-Fi test results. Wi-Fi uses least squares lateration. For lateration, the raw RSS data obtained through the measurements from three different access points are used. For least squares lateration, the raw RSS data are fitted by least squares, and the estimations of RSS data were used. A 36 m^2^ area (6 m × 6 m) was selected as the test bed for Wi-Fi, and wireless modems were placed at the three corners of the test area. The test area was then divided into 40 cm × 40 cm sections, and the corners of these sections inside the test area were identified as 196 (14 × 14) measurement points. For least squares lateration, the three access points were placed at the coordinates (0 m, 0 m), (6 m, 0 m) and (0 m, 6 m). The results for least squares lateration algorithm for the Wi-Fi were obtained with a 1.39 m average error and 35.20% accuracy for 0 to 1 m.

At the second test phase, we installed the Bluetooth low energy beacons. We used the radio frequency fingerprint method, which uses a set of location-dependent radio frequency signal parameters, such as RSSI. In most applications, even small changes lead to an impairment in the mapping between a fingerprint and a certain position. Therefore, the correlation database (CDB), which is a collection of fingerprints, needs to be updated for each new application. Fingerprints are collected during the training phase and stored in the correlation database. Each fingerprint is associated with a unique set of indoor coordinates. We use search space reduction techniques, which include a two-level filtering of received data with respect to CDB. The first level filtering compares the maximum RSSI of the received data with the maximum RSSI values of the CDB in order to minimize the search area. The second level filtering further minimizes the search area by comparing the RSSI ordering of the received data and the reference data. It is possible to increase the number of these steps as more variables are added to the CDB. To find the maximum correlation and minimum difference between received and reference values to match to a grid, three different matching algorithms are implemented. These are absolute differences matching, Euclidean distance matching and Spearman rank correlation matching. The best results were obtained with Euclidean distance matching [36]. It should be noted that no further estimation or smoothing algorithms are used. We have ongoing research projects implementing genetic algorithms based on a natural selection process that mimics biological evolution. Both methods repeatedly modify a population of individual solutions.

As the test bed for the BLE beacons, a 37.44 m^2^ area (9.6 m × 3.9 m) was selected, and the BLE beacons were placed between the grids. There were 12 grids (4 × 3) in the test bed. Each grid size was 2.4 m × 1.3 m. The BLE beacon advertising period was 800 ms, and the scan period was 200 ms. Two-and-a-half minutes of training data for each grid were used to form a reference correlation database with an average RSSI for each BLE beacon in each grid. The results for the genetic algorithm with Euclidean distance matching of BLE beacons had an average error of 0.86 m and 75.73% accuracy for 0 to 1 m. 

Our third, and final, test bed was installed for the UWB sensors. With this technology, we used three anchor devices (transmitters) and a tag device (receiver) for implementation. It was necessary to obtain the sensitive positions of each anchor device and the ToF of the radio package between the tag and anchor devices to calculate the exact indoor location of the tag devices. When N pieces of ToF information belonging to a tag device are received, we use the trilateration method to calculate the exact position of the tag device. It is possible to consider the trilateration problem as an optimization problem, and we can solve it with the least squares method. We used the Levenberg–Marquardt algorithm [51] to obtain the result. In general, we need to calculate the distance value as in the trilateration algorithm or to calculate the time as in the ToF algorithm. Thanks to the Levenberg-Marquardt algorithm, it is sufficient to count the clock pulses alone to determine the distance or to calculate the time taken.

**Figure 1:**
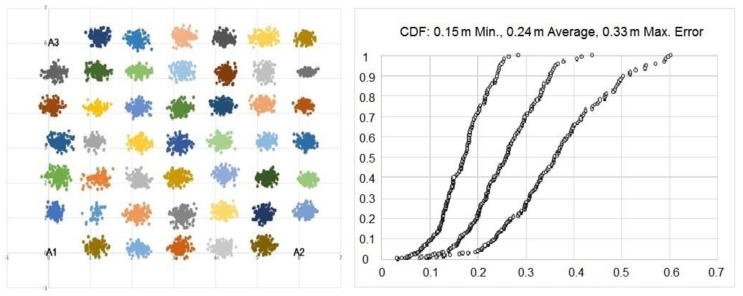
Test results of ultra-wide band sensors in a 36 m^2^ (6 m × 6 m) test area for 6900 measurements and the cumulative distribution function (CDF) of the test points of minimum, maximum and average localization errors (in meters) determined by the UWB positioning system.

The test bed for the UWB signals was a 36 m^2^ (6 m × 6 m) area. Anchor devices were placed at the corners of the test area. The UWB sensor kits used in this study spanned six RF bands from 3.5 GHz to 6.5 GHz [52]. The anchors and tags used in our test bed supported Channel 2 (4 GHz) or Channel 5 (6.5 GHz), and all tests were performed at 6.5 GHz. The test area was then divided into 1 m × 1 m areas, and the corners ((0 m, 0 m), (0 m, 6 m), (6 m, 0 m)) of these areas inside the test area were identified as 49 (7 × 7) measurement points. No measurement was taken for the points that were occupied by three anchors, resulting in 46 measurement points for the UWB test area. We conducted 150 measurements in each of 46 points: a total of 6900 measurements (see Figure 1) and obtained an average error of 0.24 m in total.

The test results for the trilateration and ToF with the Levenberg–Marquardt algorithm of UWB sensors were obtained as a 0.24 m average error and 100% accuracy for 0 to 1 m. UWB technology predicts indoor location better than the other tests conducted with BLE beacons and Wi-Fi technology, which yielded average error of 0.86 m and 1.39 m, respectively. As shown in Table 3, the accuracy (error ranges) and average error of UWB technology is much better than the other two technologies.

**Figure 2:**
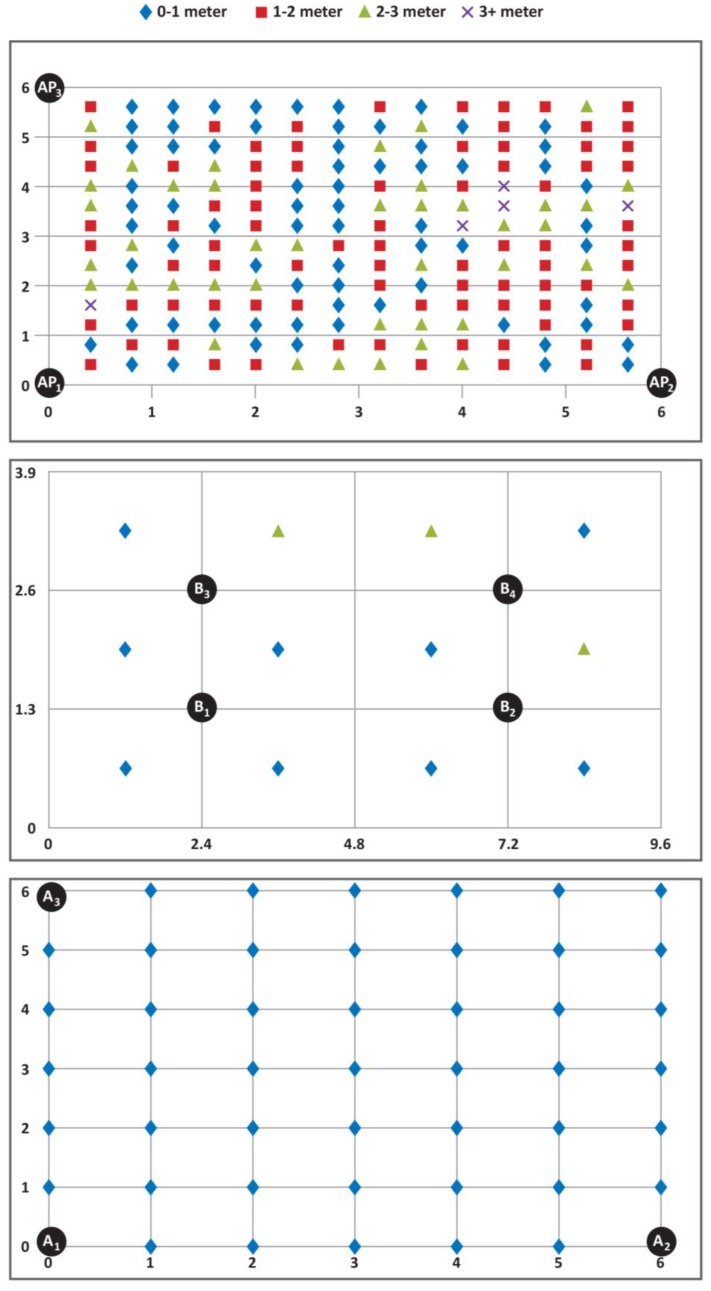
Reference points’ accuracy errors for Wi-Fi, BLE beacon and UWB (from top to bottom). AP_x_: wireless access points; B_x_: BLE beacons; A_x_: UWB anchors.

In Figure 2, the accuracy errors in determining the reference points using Wi-Fi, BLE beacon and UWB sensor technology are given. In a Wi-Fi test, the number of the reference points with an accuracy error of less than 1 m is 69, so this is 35.20% of a total of 196 reference points. In the BLE beacon test, the number of the reference points with an accuracy error of less than 1 m is nine, so this is 75.73% of a total of 12 reference points. In the UWB sensor test, the number of reference points with an accuracy error of less than 1 m is 46, so this is the total number of reference points. Thus, applying the UWB sensor technology to indoor positioning significantly improves the performance of indoor positioning.

It should be noted that in all three tested technologies, a single transmitting sensor is worn at the chest level. Thus, the placement of the sensor on the body is held constant during all experiments controlling any bias due to this factor. When there is more than one transmitting sensor to be positioned, placement of the sensor on people is even more crucial since it provides the orientation of people and affects line of sight. 

These three tested technologies have never before been assessed for their applicability for indoor behavior mapping. Generally, in the indoor positioning literature, these technologies have been tested in a given scenario against a predetermined well-defined problem. Thus, previously, the accuracy of measurements were considered only in terms of those that worked best in scenarios that were very limited when compared to the complex behavioral information that can be gathered via behavioral mapping. Understanding human behavior through their locations requires evaluating their proximities to other people and objects, rather than providing solutions to a location-based problem.

## 3. The Use of an Active Learning Center Behavioral Dataset to Simulate Tested Technologies

**Figure 3:**
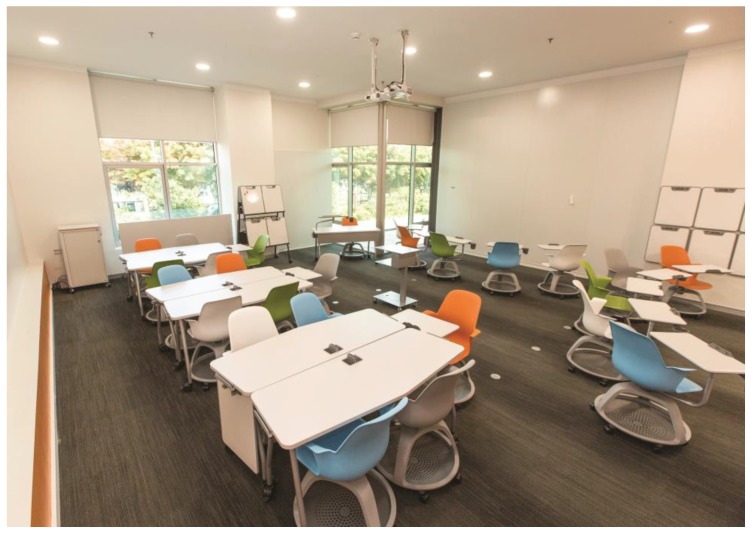
Active learning center (ALC) configuration supporting group studies.

To simulate how tested technologies would work for behavior mapping, we used a real behavioral dataset collected from a classroom. The context of the observation is a state-of-the-art active learning environment, which is a classroom furnished with moveable furniture, providing multiple choices for seating. The walls are clad with ceramic-steel surfaces that can be used for projection, writing or displaying posters by attaching magnets. The classroom also has a table for the presenter, a console for a projector (in addition to two fixed projectors in the ceiling), a caddy for hand boards and a small storage unit, all easily moveable. Located at the ground floor of a building on the campus, the classroom is easily accessible from the main entrance and opens up to a social area. Designed for small classes, the capacity of the classroom is limited to 28 people and space is designed to provide maximum control to the users; it has individually operable lights, HVAC and windows. There are multiple outlets on the floor and around the baseboard, supporting a flexible use of the environment while working on laptops. All of these design features are expected to act as affordances and support students’ use of all locations in the classroom serving their different behavioral patterns. With these features, every seat in the active learning center (ALC) is a “good seat”, and the configuration of the furniture can be changed in a matter of minutes to accommodate all types of classes (Figure 3).

The ALC behavioral dataset was collected during a three-hour workshop. There were 19 students and one professor in the classroom during observations. All participants were free to go in and out of the classroom at any time during the workshop. In addition to 13 observation cycles, multiple photos were taken from different angles. We opted for a 15-min interval between taking photos in order to capture the changes in the room objectively without disturbing the participants and the natural flow of the workshop. Later, these photos were used to produce each observation on a plan layout for each of the observation cycles. Changes in the location of the furniture were carefully updated in all observations. An observation cycle contains people observed at the time of observation along with their indoor locations. All 13 observation cycles were overlapped to make an overall map containing 247 observations where each observation is an individual. The process of obtaining an overall map took nearly 40 h of work, excluding the time spent for the preparation of the groundwork for this research. 

**Figure 4:**
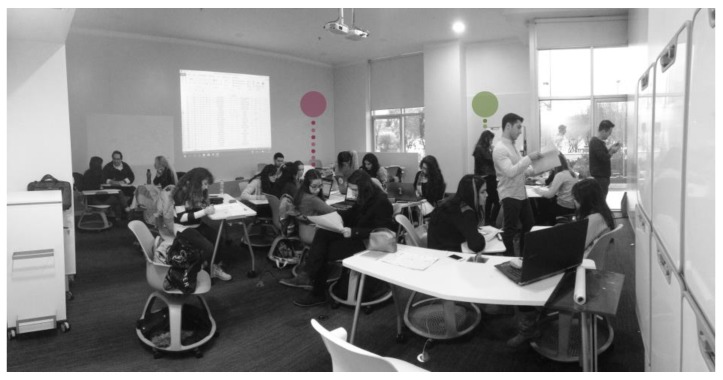
A photo from the workshop indicating randomly-selected participants for simulation.

**Figure 5:**
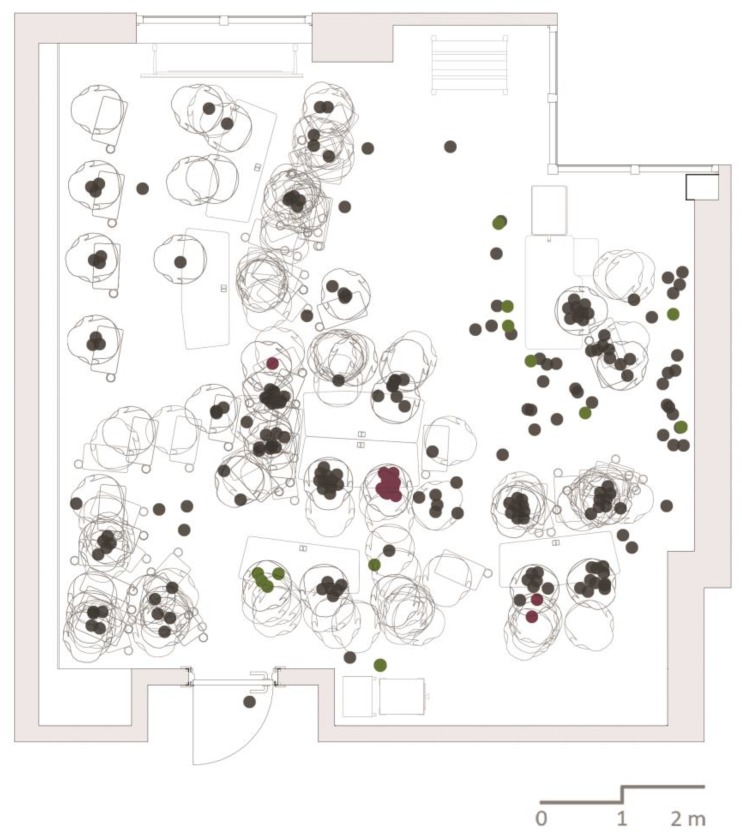
Overall behavior map of the observed workshop indicating observations of selected participants for simulations.

After manually creating the overall map, we randomly selected two participants marked with the colors red and green, as shown in Figure 4 and Figure 5. We isolated locational data related to these participants to create a simulation of how these locations would be visualized if their data were collected by any one of the three tested indoor positioning systems. The locations of the participants were not fixed since, in line with the requirements of the workshop, the students needed to use writing surfaces, work on media projected on the walls, enter the data into their laptops and work with other students.

The isolated data showed that one of the participants was more active (shown as green) than the other (shown as red), and she was either standing or moving around most of the time (Figure 5). The other participant, on the other hand, was seen sitting during all observation cycles. Through the isolated data pertaining to these two, a special overall map was produced. In order to further discuss whether the simulations we made can provide any information besides the location, the furniture used by the participants was also marked on this map.

**Figure 6:**
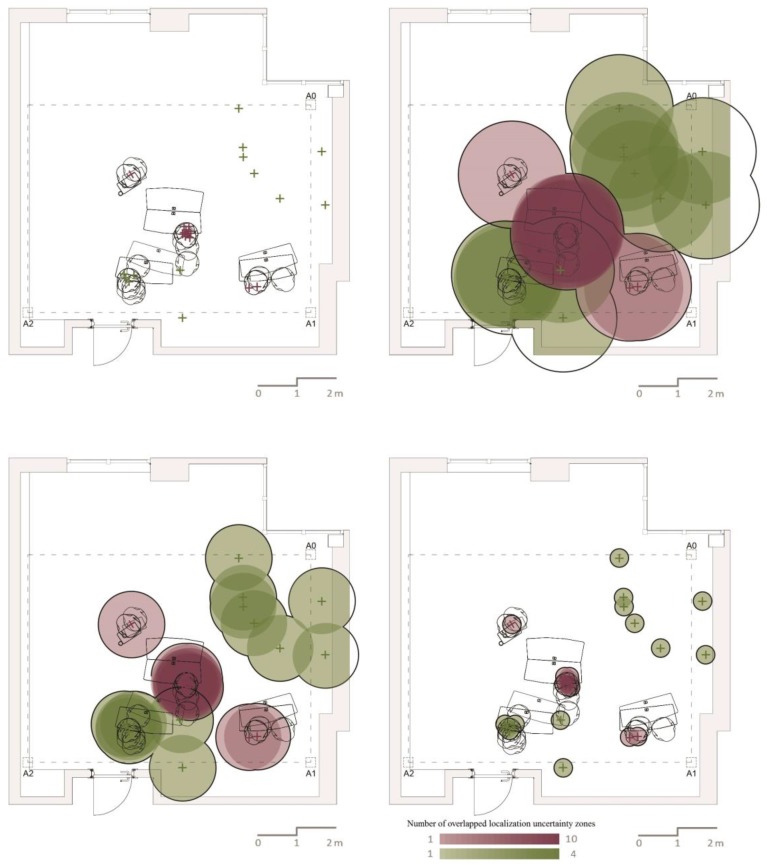
Simulation of observations using the three tested technologies. This simulation was made by incorporating the real locations of two students selected from a behavioral dataset collected from an active learning classroom during a three-hour workshop. From left to right, top to bottom: real locations, Wi-Fi, BLE beacon and UWB.

As shown in Figure 6, the simulated maps are created by applying localization uncertainty zone of the accuracy measures obtained by testing each of the three aforementioned technologies. The plus sign represents the observed locations of the two students selected, and the circle around each location represents the localization uncertainty zone measured by each tested technology. Each student’s location is represented by a transparent color. When the localization uncertainty zone (measured accuracies) of each observation overlap, then the colors get darker. In low accuracies, the circles are larger, reducing the chance of understanding discrete locations. The green represents a mobile student, whereas the red represents a static student. As the signals are transmitted in circular patterns, these localization uncertainty zones show the area in which real indoor positioning can be located. The localization uncertainty zone for Wi-Fi is a circular area with a radius of 1.39 m within a 6.07 m^2^ space. For the 0.86 m average error of the BLE beacon, the area of the localization uncertainty zone is 2.32 m^2^, covering 2.6-times less area accurately than that of the Wi-Fi. Finally, the area for the UWB sensors with a 0.24 m average error is 0.18 m^2^, far smaller than the localization uncertainty zone of the Wi-Fi or BLE beacon.

When the maps produced based on these three scenarios utilizing each of the three technologies are examined, we see that they look similar to the photographs of different resolution levels. As photographs of low, medium and high resolution serve different purposes, indoor positioning performed through employing Wi-Fi and BLE beacon technologies may be beneficial for scenarios other than behavior mapping. However, the complex task of behavior mapping requires a highly accurate location positioning that cannot be achieved by these two technologies.

It should be noted that the data used to visualize these three scenarios are based on observational data collected from an actual environment. In other words, if we had used the actual hardware of the tested technologies, there would also be an offset from the captured signal due to the placement of the devices on the participants’ body. In Wi-Fi and BLE beacons, this would have worsened the already low average error. However, with a 0.18 m^2^ localization uncertainty zone, the third technology, the UWB, would fall right into the personal spaces of the people observed.

All of the technologies tested provide information on the mobility of the participants through tracking the changes in the (*x*, *y*) coordinates of the signal. However, UWB is the only technology that captures *z* coordinates, among tested technologies: this means that, based on the placement of the wearable device, it is possible to detect whether the person being observed is sitting or standing. Based on our tested accuracies and simulations, the best technology for behavior mapping is UWB for three main reasons. First, the map produced by the simulated data and behavior map of the real data is almost identical. Second, both maps clearly indicate a relationship between the observed people and their immediate environment. Third, even when there is a limited change in location, separate observations can be recognized, staying within a very small localization uncertainty zone.

Based on the selection of the technologies we tested for automated behavior mapping, one might claim that the ranking of measured average errors was obvious from the beginning. However, these tests are not being carried out to rank average errors, but to simulate them for their suitability in understanding human behavior through location. The occupancy of space happens in a personal space that is a sort of invisible bubble surrounding us, and social interaction happens at an interpersonal distance that can be measured by the proximity between, and orientation of, different people. If the BLE beacon and Wi-Fi technologies were more accurate in our tests when used with their regular settings, then they might have some potential for behavior mapping by providing information on the use of space and proximity of people. In Figure 6, the occupancy of space by two people intersect and become blurred in BLE beacon and Wi-Fi simulations when we applied measured average errors as the localization uncertainty zone. Yet, discrete locations are nearly readable in the UWB simulation allowing one to understand the use of space by each person. Finally, as we continue our own research employing UWB sensors, the broader research community can make use of the presented simulations for their adaptations of other not-so-accurate technologies, such as BLE beacons and Wi-Fi.

## 4. Test Setup for the Realization of Behavior Mapping of an Active Learning Classroom

We set up a 7.35 m × 5.41 m test area in an active learning classroom where we collected a behavioral dataset that we used for the simulations presented earlier in this paper. We also established a ceiling system that held anchors on the exact corners of the testbed at a constant height of 2.85 m. The ceiling system was developed to provide better line of sight and direct path between the tags and the anchors. A decaWave (Dublin, Ireland) DW1000 kit was used to conduct this experiment, by incorporating three anchors in the developed ceiling system and two tags for participants. 

**Figure 7:**
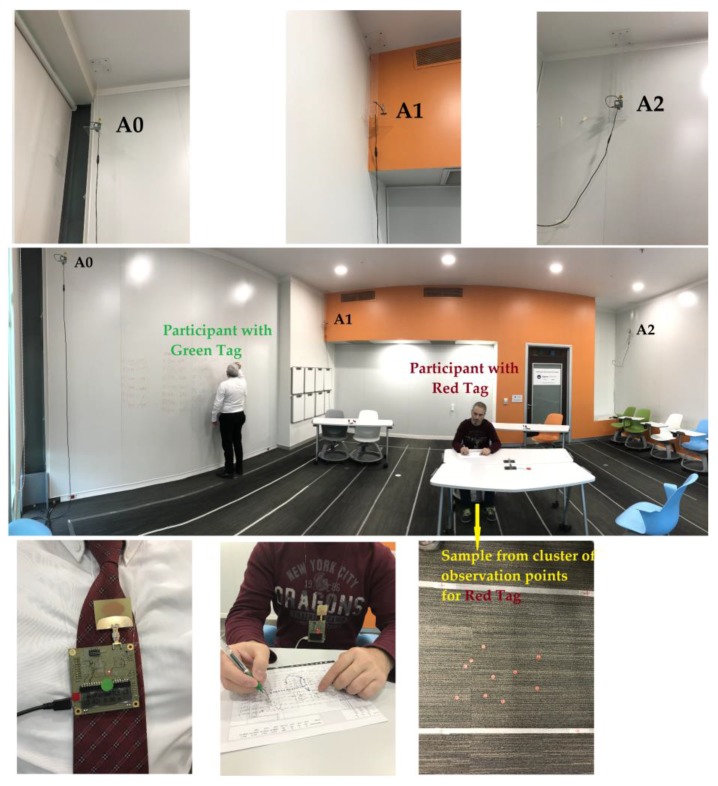
Introduction of the custom-designed ceiling system, the test bed setup with two tags (red and green), three anchors (A0, A1 and A2) and marked locations for each participant wearing a tag.

We marked 26 locations of two people on the floor and used green and red dots to identify 13 locations for each person. These locations were exactly the same as the ones we had used for the simulations presented earlier. Each test participant was given a UWB sensor tag to wear around his/her neck at chest level, and then, we collected the location data of two people to replicate the observations used in the simulations. Green and red colors were also assigned to tags for easy identification and to keep consistency in representation throughout the study. The furniture associated with observed behaviors were also set up as they were in the original observations. The ceiling system, test area defined in the active learning classroom, participants with tags and sample of cluster of observation points can be followed in Figure 7.

Both participants stayed together in the testbed for three minutes for each location providing 180 samples for each marked location adding up to a total of 4680 (26 test points × 180 samples) location measurements collected. Three points, all from the green tag, were out of the test area, but we still included them in our tests to understand how the UWB system behaves for points that are outside of the test area.

**Figure 8:**
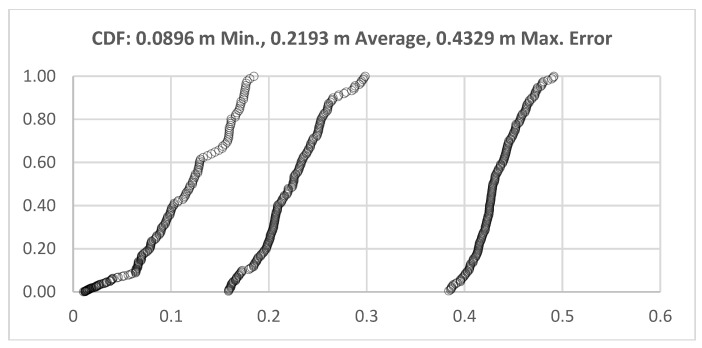
Behavior mapping test results of ultra-wide band sensors for 4680 measurements, the cumulative distribution function (CDF) of the test points of minimum, maximum and average localization errors determined by the UWB positioning system (23 test points inside; three test points outside, the test bed; and 180 measurements per point).

The mean average error measured for each marked locations are given in Table 4. Thirteen test points were selected for each participant, so there were 26 test points available in the test. The locations of each test point were given by its *x* and *y* values, and the average error was calculated. Twenty-three test points were inside the test area out of 26 test points. The average error of these 23 tests points was 0.2072 m. On the other hand, the average error of the total of 26 points was 0.2193 m. We obtained a 0.0896 m minimum error value and a 0.4329 m maximum error value. The cumulative distribution function (CDF) of the test points of minimum, maximum and average localization errors determined by the UWB positioning system are given in Figure 8.

**Figure 9:**
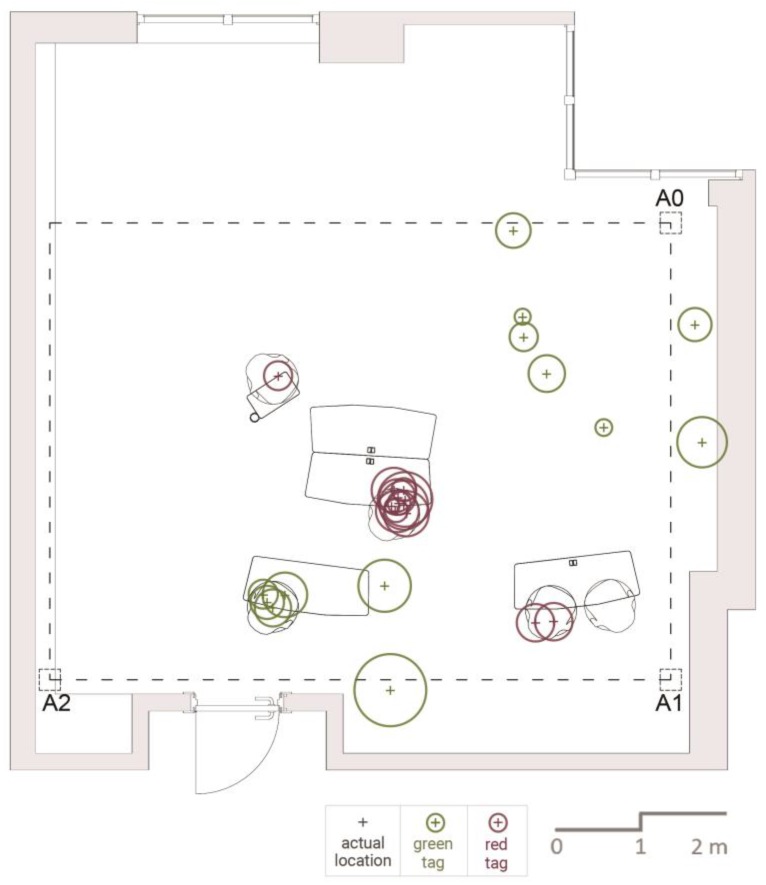
Plan layout showing mean average errors based on new UWB sensor data collected from 26 locations inherited from the simulations. One-hundred eighty location data per point were collected and added up to 4680 measurements. Note that three points fell outside the test area due to the shape of the room, the projection of the ceiling system where anchors were placed and accompanying furniture.

As shown in Figure 9, the mean average errors calculated for each location are drawn on the plan layout as localization uncertainty zones. Within the measured average errors, note that the location of the participants is easily noticeable and provides behavioral information. For the participant with the green tag, he was mobile and located in different parts of the classroom, used the board and sat on the chair. Although the average errors of the three locations that fell outside the test area are less clear, they still provide meaningful information. The participant with the red tag sat in two different locations for all observations, and his location fell right on the chair. 

## 5. Behavior Mapping Demo

We later reconstructed the observation cycles by replicating behaviors. The mobile student first walked to the board, wrote something and then walked to the opposite side of the class to sit on a table. He sat for a short time, walked slightly back to the table, and stood there.

**Figure 10:**
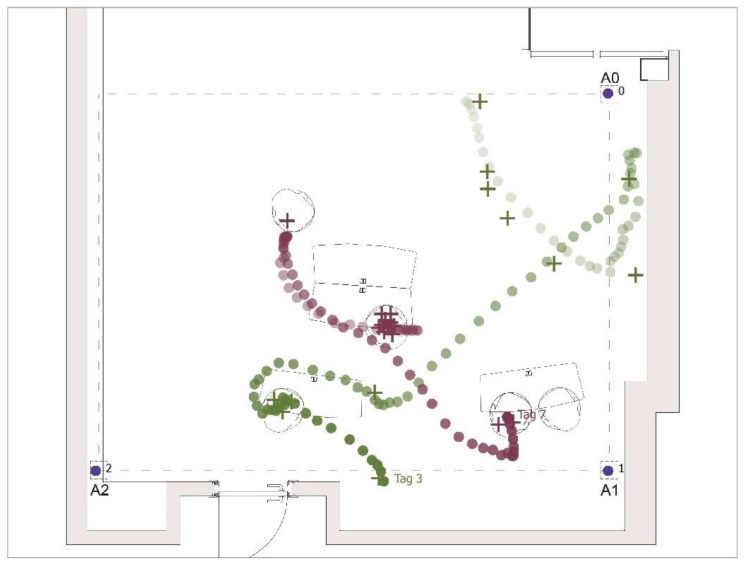
Behavior map representing the live capture of the location of two people captured by two tags and three anchors placed on a custom-developed ceiling system.

The static student sat most of the time, but changed their location to move from one table to another to sit down. Figure 10 shows that these locations and the movements to these locations were clearly captured via UWB sensors. Tracked movement of the mobile and static students are represented by the green and red dots, respectively. 

Readily available indoor positioning software is limited to displaying the read locations on screen to last 99 measurements. When the measurements exceed 99 in quantity, the last 99 remained on the screen. The locations read from each tag were each represented by a different color and gradually faded out as new readings appeared. 

We captured the screen while two subjects followed the scenarios described above. All the scenarios described above can be clearly followed in the behavior map, where one can follow the movement and locations of the participants with ease. Based on these locations, one can also estimate the set of probable behaviors associated with standing in front of the board (writing, presenting, etc.) and sitting (studying, writing, reading, etc.). In a whole class, the collected location information of these two students provides enough information to understand in which activities they were involved. Tracked routes of these two students crossed, but they passed the same locations at different times (note the difference in the intensity of the colors). If they have had stopped facing each other and had spent some time, this would have been estimated as a social interaction. From a socio–environmental perspective, location provides more than the position and an indicator of behaviors. When all people in the active learning classroom wear UWB sensors, the social interaction among peers, social interaction between the professor and the students, group formation patterns and the duration of the interactions can be estimated. Moreover, the use of resources in the classroom that were included to improve participation (including furniture, wall-to-wall board system, hand boards, etc.) can be tracked. The collected data of all sensed activities will inform all parties involved in the design and use of these environments.

While indoor positioning software is powerful in capturing locations as coordinates, it is limited in the visual display and analysis of the collected data. Our future work will incorporate the use of the geographical information science and associated software for visualization and analyses. We will also include more than two tags to be used simultaneously and both give them to people and attach them to objects. 

## 6. Discussion

To be able to make sense of the significance of the behavior mapping methods utilized in the present study, we need to revisit the core concepts and methodological issues of traditional behavior mapping (with pencil on a paper or with stylus on screen) for the automated version. Traditional behavior mapping relies on observation cycles with equal time intervals. Different sources suggest different intervals ranging between 15 min to an hour for each observation cycle depending on how frequently changes in behavior occurs [3,4,20]. The main aim is to cover a day, or a whole activity (such as a class or a play break). Statistically, based on what happens in between cycles, observations from equal intervals may not be representative samples for the whole day or an event. Although activities that have less of a chance of a regular occurrence are recommended to be observed separately, it may be difficult to keep tabs on these events, especially indoors where observers quickly record data and leave in order not to interfere with the scene. When multiple observers are involved in data collection, inter-rater reliability becomes a problem. Moore and Cosco explained that, outdoors, two observers may cover large areas by starting from the same location and walking clockwise and anti-clockwise directions [4]. Similarly, indoors, multiple observers can cover large areas. In the time spent moving over to an area after finishing observations in another area, the behaviors in the non-observed area change, and this creates a bias that has, so far, always been considered to be unavoidable. 

In automated mapping of behavior, there are no observers, but sensors that can communicate the location of the participants at timed intervals. All sensors act unobtrusively, but always present observers that are programmed to work together in harmony. It is possible to program these sensors to emit signals at any chosen time interval. Similar to the demo conducted in this study, it is also possible to program sensors to continuously emit signals. This would completely change the notion of the overall map that is produced since such a map would be a complete representation of all the sensed locations on a map rather than one created by a compilation of behavior maps in which only a limited number of observed locations are mapped. When all locations are sensed, the notion of an “observation cycle” becomes obsolete. That kind of dataset will serve as big data when the location changes of non-fixed and semi-fixed spatial features and ambient features of environment are also collected.

It is also possible to attach sensors to furniture and other objects in order to track their locations. For the presented case, if sensors are placed on furniture and environmental features in the ALC, where we collected our observational data, their positions will be automatically captured. These coordinates can later be digitally inserted onto the plan layout for representation. Furthermore, this information can be shared dynamically with users for direct feedback. In an ALC, students would know their participation levels through their use of space.

The automated behavior mapping not only solves nearly all of the methodological issues, but it also changes the terminology and concepts associated with the method. Obviously, the observers’ presence and people’s knowledge of being observed are two known factors that cause bias in the observational research. Yet, in automated behavior mapping, observed people are not natural research participants; instead they are people who are given wearable devices to actively participate in the research to provide data. Thus, from the participant’s perspective, beyond helping the researchers for the greater good, there is a good chance that they will receive immediate feedback on their tracked performance thanks to the interface that will be designed as a part of the future research process.

## 7. Conclusions

In this paper, three popular indoor positioning technologies have been considered for automated behavior mapping. Based on the tests conducted and the accuracy levels obtained in parallel with the literature on indoor positioning systems, UWB sensor technology was selected to be further studied for automated behavior mapping. Then, we reconstructed a new test area incorporating a custom-designed ceiling system. We collected new location data by marking locations used in the simulations for these three tested technologies. Finally, we provided a demonstration of automated behavior mapping by capturing the live locations of two participants using UWB sensor technology. 

The use of UWB sensor technology for behavioral mapping is a ranging problem, not a communication one. UWB technology, usually used for communications, must be set up differently to identify locations [53,54]. In a communications setup, through fine-tuning the delay spread and energy loss in the signal, emphasis is put on maximizing data speed and availability. However, behavior mapping being a ranging problem, we placed more focus on the accuracy and range. In order to understand the challenges and limitations imposed by the multipath environment, characterizing the probability of blockage and the error in the presence and absence of the direct path (DP) must be provided. One of the solutions for the reception of a direct path signal requires the placement of sensors to an optimized height [52]. When anchors are placed at an optimal height, the probability of a better line of sight (LOS) connection will increase, while the probability of the absorption of a direct path signal will decrease, both of which minimize the negative impact on accuracy through multipath positioning.

The potential of lifting sensors to an optimal height to provide better reception of sensors is not a new discovery [28,29]. Our reported efforts included multiple users in a test environment that incorporated a ceiling system with an optimized height level for anchors to maximize the line of sight and minimize the absorption effect of the human body. The optimization of the number of anchors and their height remains as a technical problem in designing any system that improves the line of sight and, thus, reduces multipath error. 

When applied widely, automated behavior mapping will improve the user experience and serve as an innovative service, even just providing locations. The transition for accommodating automated behavior mapping will be less of a challenge for organizations that are already utilizing mobile apps, badges and other wearable devices to track their users’ spatial experiences. In the near future, mobile phones may include UWB sensors that would even ease behavior mapping of indoors by eliminating the use of wearable devices [26,55]. High precision Wi-Fi, BLE beacon-based, hybrid or any future indoor positioning systems can also be applied to the behavioral mapping approach when they meet the required accuracy. A database of automated behavior maps of buildings of the same type will also serve as a knowledge base for the design community, facility managers and, most importantly, the people who are daily users of these spaces. When spatial experiences become available through automated behavior maps, they will empower users, organizations that invest in the design of these buildings and the architectural community that has been hesitant in adopting behavioral research in the design process. Ultimately, automated behavior maps will become a new way of assessing the success and failure of the built environment through a multidisciplinary approach.

## Figures and Tables

**Table 1:** Comparison of behavior mapping (BM) and real-time location systems (RTLS).

Method	Challenges	Opportunities
**Behavior Mapping**	Time and resource consuming; reliability problems; manual; limited apps	Detailed information relates location to design (built environment)
**Real-Time Location System**	Varies in accuracy based on technology used; prone to interference	Automated; various alternatives; easy to implement with new technology

**Table 2:** Comparison of selected indoor positioning technologies.

Technology	Infrastructure	Hardware	Weaknesses-Threats	Strengths
**Wi-Fi**	Uses building’s existing infrastructure	Access points, mobile	Low accuracy	Low cost; easy to implement, calibration is not necessary; no need for extra hardware
**BLE Beacon**	Dedicated infrastructure required	BLE beacons, mobile	Difficult to calibrate each BLE beacon; need extra hardware, medium accuracy; requires a greater number of cells for better accuracy; prone to radio interference	Easy to setup; easy to operate; inexpensive; low energy consumption
**UWB**	Dedicated infrastructure required	Anchors, wearable sensors	Need extra hardware; expensive compared to other technologies	Central calibration; high accuracy positioning even in the presence of severe multipath; does not interfere with existing RF systems

**Table 3:** Comparison of accuracy results.

Method	Accuracy for 0 to 1 m	Accuracy for 1 to 2 m	Accuracy for 2 to 3 m	Minimum Error	Maximum Error	Standard Deviation	Average Error
**Wi-Fi**	35.20%	41.33%	20.92%	0.05 m	3.51 m	0.72 m	1.39 m
**BLE Beacon**	75.73%	13.47%	4.93%	0.00 m	2.91 m	1.10 m	0.86 m
**UWB**	100.00%	0.00%	0.00%	0.15 m	0.33 m	0.04 m	0.24 m

**Table 4:** The mean average error of 180 measurements per test point, with a total of 4680 (26 test points × 180 samples) measurements from 26 test points for ultra-wide band sensors. The mean average was reported as 0.2072 m, including 23 test points inside the test bed, and 0.2193 m, including all 26 test points.

**Green Tag**									
**Test Point Number**	***x***	***y***	**Average Error (m)**	**Min. *x***	**Min. *y***	**Max. *x***	**Max. *y***	**Mean *x***	**Mean *y***
1	5.48	5.31	0.2101	5.6219	5.2820	5.7489	5.3657	5.6879	5.3320
2	5.60	4.30	0.1005	5.5439	4.2724	5.6684	4.4547	5.6007	4.3854
3	5.61	4.06	0.1742	5.7336	3.9189	5.8042	4.0188	5.7604	3.9744
4	5.88	3.62	0.2191	6.0387	3.4531	6.1440	3.6767	6.0884	3.5691
5	6.56	2.98	0.1052	6.4261	3.0260	6.6562	3.0960	6.5337	3.0640
6	3.96	1.12	0.3165	3.8702	0.4980	4.1510	1.1555	4.0197	0.8231
7	2.53	1.01	0.1777	2.5639	0.6792	2.6483	1.0514	2.6178	0.8734
8	2.57	0.92	0.2021	2.5741	0.6181	2.6831	0.8364	2.6358	0.7312
9	2.64	0.85	0.2232	2.6058	0.5309	2.7248	0.8004	2.6635	0.6304
10	2.78	1.00	0.2695	2.8848	0.7262	3.0004	0.9207	2.9524	0.7948
11	7.72	2.81	0.2994	7.3400	4.0374	7.9106	4.4092	7.6788	4.3201
12	7.64	4.20	0.2027	7.7133	2.7958	8.0817	3.1790	7.8698	3.0514
13	4.04	−0.13	0.4329	4.0472	−0.6171	4.1280	−0.5137	4.0938	−0.5591
**Red Tag**									
**Test Point Number**	***x***	***y***	**Average Error (m)**	**Min. *x***	**Min. *y***	**Max. *x***	**Max. *y***	**Mean *x***	**Mean *y***
1	2.71	3.60	0.1727	2.7441	3.4101	2.8458	3.5205	2.7876	3.4486
2	4.08	2.24	0.2720	3.8532	1.7055	4.2356	2.4234	4.1734	2.0008
3	4.19	2.24	0.1477	4.2990	2.0291	4.3396	2.3070	4.3112	2.1956
4	4.12	2.16	0.2158	4.2437	1.8777	4.3479	2.1210	4.2949	2.0381
5	4.21	2.13	0.3263	4.4053	1.8652	4.5344	2.1505	4.4828	1.9623
6	4.19	2.07	0.2934	4.4128	1.7713	4.4632	2.0224	4.4390	1.9216
7	4.13	2.08	0.1336	4.1672	1.7454	4.2507	2.1981	4.2252	2.1064
8	4.05	2.05	0.0896	4.0171	1.8666	4.0526	2.0510	4.0316	1.9639
9	4.09	2.01	0.1443	4.0779	1.5376	4.2647	2.0248	4.1817	1.9164
10	4.11	1.99	0.2541	4.2792	1.8492	4.4120	1.9858	4.3541	1.9247
11	4.22	1.97	0.2702	4.1872	1.7236	4.5428	1.8153	4.3797	1.7694
12	5.75	0.67	0.2270	5.7826	0.3007	5.8272	0.5966	5.8098	0.4513
13	5.96	0.69	0.2216	6.0483	0.4111	6.1298	0.6740	6.1038	0.5247
**Total 23 point average inside the test bed**								**0.2072 m**	
**Total 26 point average all points included**								**0.2193 m**	
**Min.**								**0.0896 m**	
**Max.**								**0.4329 m**	
